# Novel Bioinformatics–Based Approach for Proteomic Biomarkers Prediction of Calpain-2 & Caspase-3 Protease Fragmentation: Application to βII-Spectrin Protein

**DOI:** 10.1038/srep41039

**Published:** 2017-01-23

**Authors:** Atlal El-Assaad, Zaher Dawy, Georges Nemer, Firas Kobeissy

**Affiliations:** 1 Department of Electrical and Computer Engineering, American University of Beirut, Riad El Solh, Beirut, Lebanon; 2 Department of Biochemistry and Molecular Genetics, Faculty of Medicine, American University of Beirut, Riad El Solh, Beirut, Lebanon

## Abstract

The crucial biological role of proteases has been visible with the development of degradomics discipline involved in the determination of the proteases/substrates resulting in breakdown-products (BDPs) that can be utilized as putative biomarkers associated with different biological-clinical significance. In the field of cancer biology, matrix metalloproteinases (MMPs) have shown to result in MMPs-generated protein BDPs that are indicative of malignant growth in cancer, while in the field of neural injury, calpain-2 and caspase-3 proteases generate BDPs fragments that are indicative of different neural cell death mechanisms in different injury scenarios. Advanced proteomic techniques have shown a remarkable progress in identifying these BDPs experimentally. In this work, we present a bioinformatics-based prediction method that identifies protease-associated BDPs with high precision and efficiency. The method utilizes state-of-the-art sequence matching and alignment algorithms. It starts by locating consensus sequence occurrences and their variants in any set of protein substrates, generating all fragments resulting from cleavage. The complexity exists in space O(mn) as well as in O(Nmn) time, where N, m, and n are the number of protein sequences, length of the consensus sequence, and length per protein sequence, respectively. Finally, the proposed methodology is validated against βII-spectrin protein, a brain injury validated biomarker.

Degradomics discipline has been recently introduced to depict the application of an omics approach (genomics and proteomics etc.) to identify different proteases and their subsequent proteolytic substrates/degradome in a defined pathophysiological condition^1^. Recently, the use of bioinformatics tools as means for data mining has spanned different fields in cancer, neuroscience and biochemistry research^2^,^3^. Degradomics as a discipline has benefitted from data mining strategies as tools to predict degradome specific substrates *in silico*^4^,^5^,^6^,^7^. However, the application of bioinformatic tools on degradomics analysis requires different types of sequencing matching algorithms making it one of the challenging fields despite its potential beneficial outcomes mainly in clinical and diagnostic research. Knuth *et al*. developed an algorithm that only finds exact matches of a subsequence of size m in a sequence of size n in O(m + n)^8^. It is worth to know that other algorithms have identified sequence variants with comparable complexity, but not with the same fidelity. Lipman *et al*. devised a heuristic algorithm called FAST Protein (FASTP)^9^; it is based on alignment approach and is both rapid and sensitive in finding similarities between any amino acid subsequence and matching sequences in a database. Yet, it does not cover all regions, as it starts with an anchoring scheme that identifies identical regions using a replaceability matrix^9^. Similarly, Altschul *et al*. developed another heuristic algorithm BLAST, along with its variations; this algorithm supposedly supersedes FASTP in performance while retaining comparable sensitivity^10^. Nonetheless, it also uses seeds for basic anchoring as it identifies similar sequences to the query sequence by seeking segment pairs comprising a word pair of a given score.

In contrast, Ning *et al*. devised a sequence search and alignment algorithm based on the Sequence Search and Alignment by Hashing Algorithm (SSAHA); this method performs three to four times faster than FASTP or BLAST, as it handles searches in databases of gigabyte range^11^. However, it is associated with overhead as it pre-processes the sequences in the database by breaking them into consecutive k-tuples, and then uses a hash-table to store the position of each k-tuple occurrence. Ma *et al*. devised another search algorithm that works faster than BLAST, with both a modest memory usage and higher sensitivity, covering a wider seeding model^12^. Nevertheless, it is also based on heuristics, compromising accuracy to a certain extent. Kurtz *et al*. developed a suffix tree-based method for similarity sequence search, implemented with linked lists^13^. This method performs well, but is limited to exact searches and suffers from overhead due to large space requirements, with continual and necessary updates requirements to the linked lists. Lecroq developed an algorithm based on the Q-gram hashing; it is considered the fastest so far, especially on a small size alphabet, because it searches the sequence database using an efficient indexing technique^14^. Nonetheless, it is also limited to searching for exact matches. Needleman *et al*. created the first method for biological sequence comparison based on dynamic programming^15^. Even though this method is considered to be optimal, it is based on global alignment, which renders it more specific to sequences of comparable sizes.

Applications of degradomics studies have been witnessed in several diseases, such as brain injury and cancer^6^,^7^,^16^,^17^. In brain injury field, both calpain-2 and caspase-3 proteases generate signature protein markers that would theoretically be indicative of different types of neural cell injury mechanisms^16^,^17^,^18^. These signature markers are fragment proteins or BDPs, resulting from proteases-associated cleavages. Since they are differentiated by their sequence and molecular weight (Mwt) specificity, they are considered unique to each protease with a definitive signature Mwt characterized by a well-defined amino acid sequence. Degradomics-peptidomics profiling of blood plasma, for instance, showed high sensitivity to changes not evidenced by standard proteomics techniques, providing unique signatures of diagnostic utility^19^.

In cancer research, Itoh *et al*. presented a review of the intense role of MMPs in cancer disease. Metalloproteases, MMP-2 and MMP-9, generate protein substrate fragments that are indicative of malignant growth^20^. Similarly, Fuhrman-Luck *et al*. used degradomics studies to identify kallikrein-related peptidase (KLK) substrates as biomarkers for cancer disease^21^. In Lopez-Otin *et al*. work, the local degradation of extra cellular matrix (ECM) forming the physical barrier for cell migration components is observed due to the activation of matrix MMPs^22^. MMPs, similar to other proteases (caspases, calpains and cathepsins) can truncate proteins at specific amino acid sequences^20^,^22^.

On the other hand, degradomics studies have been noticeable in the genetic aspects of congenital heart disease (CHD) since they represent major causes of birth defects in newborns. A crucial gene in this context is the *TLL1* which encodes a metalloprotease. Upon activation, this metalloprotease truncates extracellular substrate proteins in the septum and the resulting BDPs represent putative markers of the disease^23^. Moreover, degradomics studies have contributed significantly to the field of neuroscience particularly in neural injury conditions. Glantz *et al*. discerned the molecular basis of protease-catalyzed proteolysis of αII-spectrin and βII-spectrin in the different injury scenarios, indicative of different neural injury techniques (both apoptotic and necrotic)^24^.

The main potential of this work lies in its ability to predict fragments sequences computationally prior to wet experiments. This work extends on previous research in identifying brain injury specific BDPs, utilizing data of different potential substrate proteins, extracted from public databases^25^. The goal is to computationally search through the set of selected proteins for potential breakdown sites (consensus exact matches as well as variants), subject to fragmentation, and subsequently generate all possible cleaved fragments (BDPs). The work presents a dynamic programming solution based on modifications to the Smith-Waterman (SW) algorithm. Accordingly, the solution is based on local alignment and runs in time and space complexity O(mn) per protein sequence (m and n represent the sizes of the consensus sequence as well as the protein sequence, respectively). The method is applied to calpain-2 and caspase-3 proteases which are associated with the execution phase of both the apoptotic and necrotic cell death, and where the distinction between the two dominated types of cell death is crucial to better reveal the injury mechanisms.

This paper is organized as follows: We first describe the results of applying the computational method to the cleavage of βII-spectrin substrates, particularly in brain injury. It then describes the implicated proteases calpain-2 and caspase-3 as well as the distinction between the associated neural cell deaths (necrosis/apoptosis). After elaborating on the cleavage modes of calpain-2 and caspase-3, the results section presents the generated data for both the βII-spectrin protein and the mouse genome used in testing the algorithm and the corresponding output results. Afterwards, discussion of output is presented in the discussion section. Finally, the methods section defines the main problem and then describes the method and the algorithm involved in more details.

## Results

### Calpain-2 and Caspase-3 Proteases

**Figure 1:**
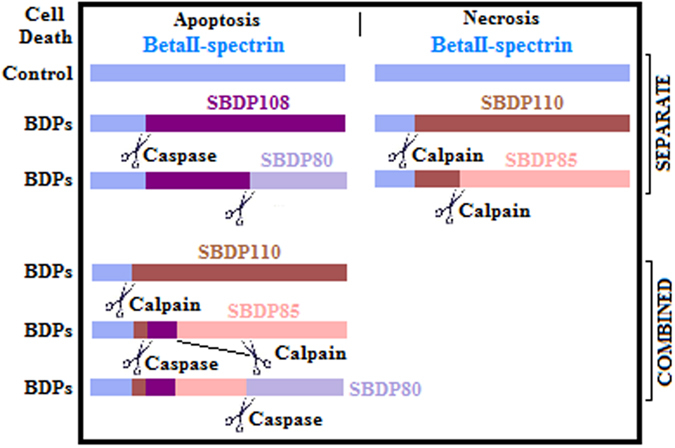
Schematic of necrosis and apoptosis cell death pathways. Figure shows necrosis type of neuronal cell death on the right, with calpain-2 specific fragments SBDP110 and SBDP85. On the left, the figure shows apoptosis neuronal cell death type, with caspase-3 specific fragments SBDP108 and SBDP80. The fragments are labeled with their approximate sizes. On the bottom, the figure shows the breakdown products by order of generation. First, calpain-2 cleaves βII-spectrin and generates SBDP110. Afterwards, and if both of caspase-3 and calpain-2 are activated, SBDP110 is cleaved by both proteases, generating SBDP108 and SBDP85. Lastly, caspase-3 cleaves SBDP85 and produces SBDP80 (apoptosis-specific)^16^,^32^.

Both calpain-2 and caspase-3 are activated in different modes of neural cell death and; thus, it is essential to characterize their spatio-temporal activation as it is indicative of the injury mechanisms. Calpain-2 protease is activated in necrosis and apoptosis; it generates calpain-specific cleaved fragments. On the other hand, caspase-3 protease is only activated in neural apoptosis and generates caspase-specific cleaved fragments. Both of these proteases cleave their associated protein substrates at a cleavage site predefined within each consensus occurrence resulting in unique BDPs. Since the BDPs are differentiated by their protease-generated molecular weight (see Fig. 1), they are specific to each protease and represent molecular signatures^16^,^18^,^26^.

In order to characterize the possible cleavage fragments of an already characterized protein, we assessed the brain injury biomarker βII-spectrin with its BDPs post calpain-2 and caspase-3 activation. We apply our proposed *Cleaved Fragments Prediction Algorithm for Calpain and Caspase (CFPA-CalpCasp*), that is specifically designed to assess calpain-2 and caspase-3 cleavage substrate sequences^25^. The algorithm is based on dynamic programming principles and is efficient in terms of both time and ‘Space Complexity’ (Run time) complexity. This algorithm is capable of performing local sequence alignment achieved via a scoring table; in addition, it is able to find consensus occurrences and variants of the consensus sequence after accounting for insert and delete operations^27^.

The algorithm CFPA-CalpCasp proved its effectiveness after validating its results against experimental studies from previous works in the literature. This allows the utilization of the proposed computational methodology to guide and complement further experimental studies. Both of calpain-2 and caspase-3 proteases can lead to different cleaved fragments depending on whether the activation is combined or separate. The following three subsections illustrate the three different cases of when caspase-3 is activated separately, then when calpain-2 is activated separately, and finally when both of caspase-3 and calpain-2 are activated together (see summary in Table 1).

### Caspase-3 Cleavage Mode (Apoptosis)

Caspase-3, similar to calpain-2, is a cytosolic cysteine protease. However, caspase-3 differs from calpain-2 in the requirement for Ca^2^. Furthermore, what distinguishes caspase-3 from other proteases is that it has a crucial role in apoptosis in many different cell types^27^. When caspase-3 is activated, it functions as a downstream mediator in apoptosis and exclusively generates βII-spectrin BDP specific fragment “SBDP108”. Caspase-3 cleaves the substrate after finding both of Asp in the first position (P1 position) and Asp in the fourth (P4 position), whereas any amino acids can occupy the second position (P2 position) and the third position (P3 position), as indicated in Table 1^18^.

### Calpain-2 Cleavage Mode (Necrosis and Apoptosis)

The association of calpain-mediated proteolysis to necrotic neuronal death has gained major research focus. This relation was revealed in ischemic and excitotoxic neural injury^17^. Calpain-2 cleaves the substrate after finding either of Val, Leu, or Ile residues in the target protein. It cleaves in the second (P2 position) after Val, Leu, or Ile amino acid is found in the first (P1 position). Accordingly, the P2 position in the target protein can be any residue (for example, Tyr, Gly, Arg) (as shown in Table 1)^18^.

### Combined Cleavages of Calpain-2 and Caspase-3

Calpain-2 protease is usually activated before caspase-3. This temporal profile allows both proteases to cleave one protein substrate at separate cleavage sites without interference. It is possible though, in a random and infrequent instance, for calpain-2 and caspase-3 to be activated concurrently. Some injury models reveal the synchronized activation of both proteases like the *in vivo* model of traumatic brain injury (TBI), which affects different areas of the brain. In addition, other neuronal injury mechanisms, demonstrating the activation of both proteases, include NMDA, kainate, and glucose–oxygen-deprivation cerebrocortical neurons^28^,^29^,^30^.

### Input Data

The algorithm needs to be validated with real data to verify its accuracy and effectiveness. Thus, the substrate βII-spectrin is used for input data, as shown in Supplementary Fig. 1. In addition, the mouse genome is also used as input data to test the efficiency of the algorithm on a large dataset^31^.

### Output of βII-spectrin Cleavage by Caspase-3

**Figure 2:**
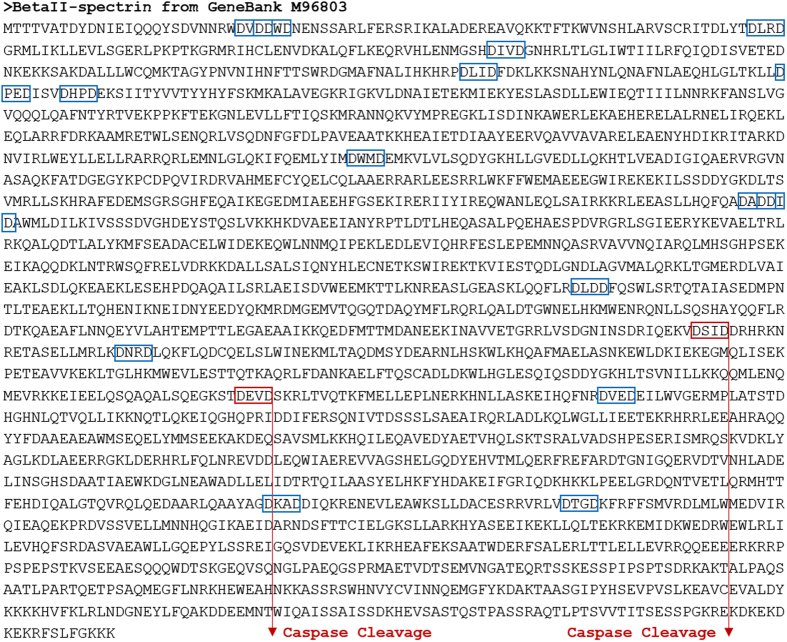
Cleavage sites of βII-spectrin by caspase-3. The figure shows all the consensus subsequences predicted by the algorithm, surrounded in boxes, and obeying the amino acid sequence DXXD (D stands for Asp amino acid and X can be any amino acid). In particular, the red boxes represent the consensus occurrences validated experimentally. In addition, the figure shows the cleavage site where caspase-3 cleaves.

The pattern DXXD↑X corresponds to the consensus sequence for caspase-3 protease, where X represents any one amino acid from the twenty primary amino acids, symbol ↑ represents the site of cleavage, and D represents Aspartic acid (Asp) amino acid. All different combinations of the above pattern correspond to 400 expected instances in total. The partial amino acid subsequences, presented in Fig. 2, highlight caspase-3 consensus occurrences showing two *hits* in red that are validated experimentally^16^,^32^. Figure 2 also shows caspase-3 protease cleavage mode in cleaving an input protein sequence substrate^25^. The results of applying CFPA-CalpCasp algorithm on βII-spectrin input protein sequence and caspase-3 protease are shown in Table 2. The table indicates all the consensus occurrences (*hits*) detected by caspase-3 protease for cleavage, including the cleavage site corresponding to each consensus occurrence. The start and end positions of each consensus occurrence within the given input protein sequence are also indicated. Furthermore, the table shows all the fragments generated from cleaving the input protein sequence, at the detected consensus occurrences and cleavage sites, including their start and end positions.

From the detected hits, we list the specific subsequences ‘DEVD’ and ‘DSID’, which are validated against the experimentally generated fragments^16^,^32^. Motif ‘DSID’ starts at position 1251 (or P1251) and ends at position 1254 (or P1254) within βII-spectrin input protein sequence. The corresponding cleaved subsequence fragments are ‘MTTT…DSID’, which starts at position 1 (P1) and ends at position 1254 (P1254), and ‘DRHR… GKKK’, which starts at position 1255 (P1255) and ends at position 2364 (P2364). On the other hand, the start position of motif ‘DEVD’, within βII-spectrin input protein sequence, is at P1454, and its end position is at P1457. The corresponding cleaved subsequence fragments are ‘MTTT…DEVD’, which starts at position 1 (P1) and ends at position 1457 (P1457), and ‘SKRL…GKKK’, which starts at position 1458 (P1458) and ends at position 2364 (P2364).

### Output of βII-spectrin Cleavage by Calpain-2

**Figure 3:**
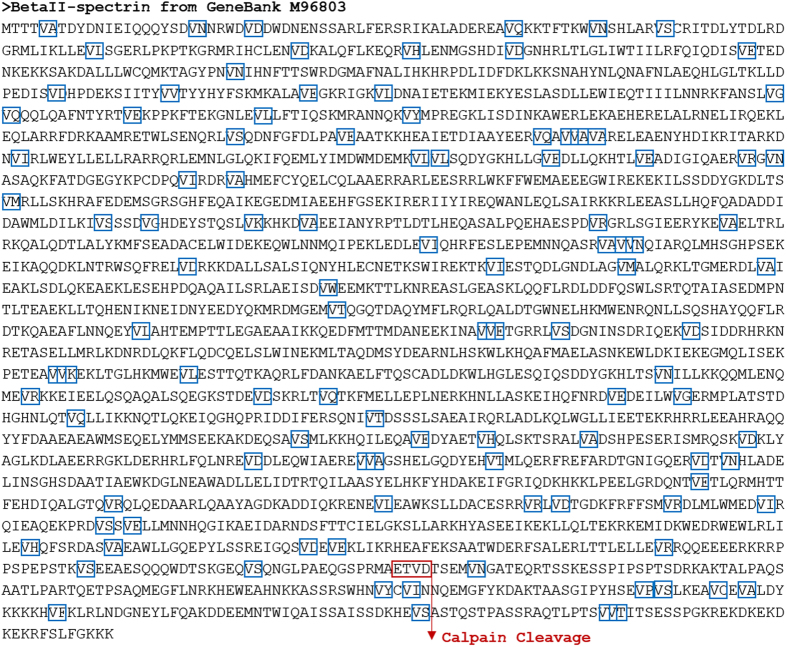
Cleavage sites of βII-spectrin by calpain-2. The figure shows all the consensus subsequences predicted by the algorithm, surrounded in boxes, and conforming with the amino acid sequence VX (V stands for Val amino acid and X can be any amino acid). In particular, the red box represents the consensus occurrence validated experimentally. In addition, the figure shows the cleavage site where calpain-2 cleaves.

The patterns of LX↑X, VX↑X, and IX↑X correspond to the consensus sequences of calpain-2 protease, where X represents any amino acid from the twenty primary amino acids, while symbol ↑ represents the cleavage site, and (L, V, I) triplet maps to (Leu, Val, Ile) amino acid triplet; respectively. All different combinations of the above patterns correspond to 60 expected instances in total. The partial amino acid subsequences, pictured in Fig. 3, highlight calpain-2 consensus occurrences, showing one *hit* in red that is validated experimentally^16^,^32^. Figure 3 also shows calpain-2 protease cleavage mode in cleaving an input protein sequence substrate^25^. The results of applying the proposed algorithm on βII-spectrin input protein sequence and calpain-2 protease are shown partially in Table 3 (see Supplementary Table 1). The table lists all the hits detected by calpain-2 protease for cleavage, including the cleavage site corresponding to each consensus occurrence, and the start and end position of each consensus occurrence within the input protein sequence. Furthermore, the table shows all the fragments generated from cleaving the input protein sequence, at the detected consensus occurrences and cleavage sites, including their start and end positions.

From the detected hits, we list the specific subsequence ‘ETVD’, which is validated against the experimentally generated fragments^16^,^32^. Motif ‘ETVD’ starts at position 2143 (or P2143) and ends at position 2146 (or P2146) within βII-spectrin input protein sequence. The corresponding generated sequence fragments are ‘MTTT…ETVD’ and ‘TSEM… GKKK’. The first fragment extends from position 1 (P1) through position 2146 (P2146), and the second one extends from position 2147 (P2147) through position 2364 (P2364). The other detected occurrences, such as ‘VH’, ‘VA’, ‘IK’, and ‘LM’ (see Fig. 3), did not appear in experimental results, and the reason could be linked to the rapid pace of the cleavage transitions. Particularly, the end-to-end cleavage sites may obscure the digestion of the detected sequence occurrences in a simultaneous manner, and thus may end up in undetectable occurrences by experimental techniques.

Simultaneous activation of both proteases (calpain-2 and caspase-3) has also been generated computationally and provides similar outcomes and insights to the separate activation of each protease. For experimental validation, two possibilities can arise in such a case: 1) one protease inhibits the cleavage of the other protease, or 2) one protease cleaves within the input sequence cleaved by the other protease.

### Output of Mouse Proteome Cleavage by Caspase-3

In order to assess the efficiency of the algorithm on a large dataset, the proposed algorithm is applied on the whole mouse proteome. Supplementary Table 2 shows all the consensus occurrences that result from the cleavage of the mouse proteome input protein sequences by caspase-3. The consensus occurrences appear per each protein sequence, including their start and end positions. Red highlights in Supplementary Table 2 cover the same consensus occurrence that appears multiple times within the same input protein sequence, including all the different start and end positions. On the other hand, blue highlights cover the consensus occurrences that overlap within one protein sequence. All consensus occurrences reflect the DXXD consensus pattern, where D represents Aspartic Acid and X represents any amino acid.

Table 4 depicts a detailed case of an input protein sequence having multiple occurrences and consensus overlaps. Supplementary Table 3 shows the corresponding cleaved fragments with their start and end positions. Nevertheless, Supplementary Table 3 lists all possible cleavage combinations of the case; Supplementary Table 3 is simplified by showing the beginning and ending of each fragment subsequence.

## Discussion

Computational prediction of biomarkers is becoming a priority for biologists, as it conserves both time and cost that would have been otherwise spent on experiments, in order to probe for biomarkers. The developed algorithm for cleaved fragments prediction (CFPA-CalpCasp) is based on Smith-Waterman algorithm and detects local subsequence similarities in a set of protein sequences^25^. Accordingly, alignments with deletions and insertions are pruned. Then, for every acceptable alignment, the protein sequence is cleaved at the predefined cleavage site within the consensus occurrence, and results in cleaved fragments identified by the algorithm. The consensus occurrence variants are built within the consensus pattern for calpain and caspase, such as in subsequence DXXD, where D is fixed for Asp, but X can be any amino acid.

To assess the effectiveness of the algorithm in identifying consensus subsequences, proteolysis, and fragment breakdowns generation, it is applied to βII-spectrin substrates. The corresponding results are validated with experimental data from the literature, demonstrating the accuracy of the algorithm. In addition, the algorithm proved its efficiency in performance by detecting all the consensus variants, cleaving them at similar cleavage site, and generating all potential BDPs with relatively low time and space complexity.

Furthermore, for a better efficiency assessment, the algorithm is applied on a large dataset (mouse genome) comprising ~30 k protein sequences^31^. Once more, the algorithm proved its performance efficiency by detecting the consensus variants, cleaving them at the cleavage site, and generating the resulting breakdown products (refer to Algorithm in Supplementary Fig. 2). Moreover, the results demonstrated the functionality of all “*different and possible”* types of cleavage combinations, in addition to the functionality of “*overlapping”* consensus occurrences (refer to Supplementary Table 2).

**Figure 4:**
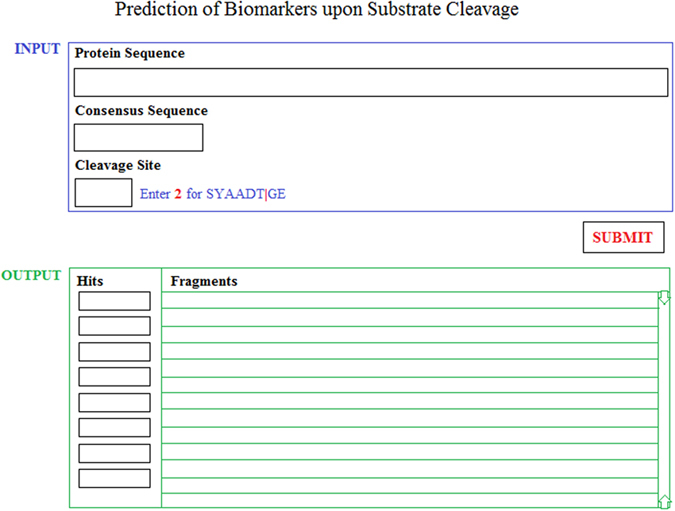
Preliminary mockup interface of the web tool. The figure shows a preliminary design for the web interface that will be developed to provide researchers with access to the proposed algorithm.

The generated data and results of this research can help guide future experiments. To make the method accessible to the scientific community, a web-based front end tool will be developed for online access by users; the application will have a database backend which will store protein substrates, relevant consensus subsequences, and the generated breakdown products (BDPs or biomarkers). The front end web interface will comprise different types of functionality. Figure 4 below presents a preliminary mockup interface of the web tool. The major functionality is that scientists will be able to select a protease, a substrate protein, and a protease cleavage mode from drop down menus; after selection of input data from the web form, they will execute the algorithm to obtain all the fragments generated upon proteases cleavage of substrate proteins. The corresponding output of biomarkers will then be presented in a list that scientists can scroll through and download for further post-processing.

Another functionality we are building into the web tool is the ability to click on a specific biomarker and output all the corresponding properties, by linking to public databases. Such information is crucial for experimentalists as it allows them to identify whether a specific biomarker has an existing antibody, instead of designing a new one. Furthermore, the above strategies can be applied to other disciplines utilizing degradomics as means for biomarker identification.

## Methods

### Problem Definition

The problem is to locate all consensus occurrences of a consensus subsequence in a set of protein sequences. Once the consensus subsequence is detected on the protein sequence, the protease enzyme can then cleave a protein substrate at the predefined *cleavage site* within the consensus subsequence. This results in the formation of fragment subsequences or BDPs, signifying disease biomarkers. The output is expected to show all occurrences (*hits*) of the consensus sequence among all input protein sequences, in addition to the corresponding cleaved fragments. The hits correspond to exact matches of the consensus model; this model also contains variants within itself (shown in Table 1 as a combination of fixed and variable amino acids).

We elaborate on the CFPA-CalpCasp algorithm in the next section. The space (Run time) complexity of CFPA-CalpCasp is O(mn) and the time (computational) complexity is O(NN’mn), where N, N’, n, and m are the total number of input protein sequences, total number of consensus sequences, size per protein sequence, and size per consensus sequence, respectively.

### Computational Method

The goal of the developed method CFPA-CalpCasp is to detect all consensus occurrences (and variants) of a specifically known consensus subsequence - with a specifically known cleavage site – in a set of input protein sequences. In addition, this method is capable to identify generated cleaved fragments upon cleavage of the input sequences^25^. Once the consensus subsequence is found (or matched) in an input protein sequence substrate, the activated protease enzyme cleaves the input sequence at the cleavage site - predefined within the consensus subsequence - resulting in fragment subsequences or breakdowns (BDPs).

Due to the specificity of calpain-2 and caspase-3 cleavage modes, CFPA-CalpCasp looks for an exact match of every stored consensus subsequence. The consensus variants are generated by fixing certain amino acids while varying others within each stored consensus subsequence. Accordingly, the *cleavage site* becomes right after the consensus *hit*. The algorithm embeds a modification version of Smith-Waterman algorithm; it performs local sequence alignments which allow to identify all local regions within each input protein sequence that match a particular consensus sequence^33^. The alignments are based on dynamic programming technique which constructs a scoring table, but then removes all consensus occurrences with INserts or DELetes (INDELs). To process N input protein sequences, the algorithm executes N times.

If multiple occurrences of any consensus subsequence are found in one protein sequence, the proteases might cleave at the cleavage site of each consensus occurrence. Consequently, all different combinations of potential cleavages are possible because each combination is actually a possible cleavage incidence by nature. Accordingly, the fragment generation module, within the developed algorithm, generates different scenarios of output fragments based on the different combinations of consensus occurrences. The following represents an illustration of the different output fragments generated by the algorithm upon detecting two consensus occurrences in one protein sequence:

- The algorithm generates the output fragments after cleaving the input protein sequence at the cleavage site of the first occurrence, resulting in fragment 1 and fragment 2.
- The algorithm generates the output fragments after cleaving the input protein sequence at the cleavage site of the second occurrence, resulting in fragment 3 and fragment 4.
- The algorithm generates the output fragments after cleaving the input protein sequence at the cleavage sites of the first and second occurrences, resulting in fragment 1, fragment 4, and fragment 5 (which is located between the two cleavage sites).

The last case c) results in many short fragments, compared to a few long ones from the first two cases a) and b).

Due to space limitations, we are not presenting the fragments generated from all combinations. Tables 2 and 3 show exact consensus matches and the corresponding cleaved fragments by caspase-3 and calpain-2 on βII-spectrin substrate respectively. Table 4 and Supplementary Table 3 show caspase-3 exact consensus matches from the mouse genome and the corresponding cleaved fragments. Moreover, the data shows the case of a single consensus with multiple occurrences within a single input protein sequence, including different occurrences that overlap (refer to Supplementary Table 2).

## Additional Information

**How to cite this article**: El-Assaad, A. *et al*. Novel Bioinformatics–Based Approach for Proteomic Biomarkers Prediction of Calpain-2 & Caspase-3 Protease Fragmentation: Application to βII-Spectrin Protein. *Sci. Rep.* **7**, 41039; doi: 10.1038/srep41039 (2017).

**Publisher's note:** Springer Nature remains neutral with regard to jurisdictional claims in published maps and institutional affiliations.

## Supplementary Material

Supplementary Information

Supplementary Table 1

Supplementary Table 2

Supplementary Table 3

## Figures and Tables

**Table 1:** Calpain-2 and Caspase-3 Cleavage Properties.

	Calpain	Caspase
Protease Class	Cysteine Protease	Cysteine Protease
Preferred Cleavage Site (*)	AspxxAsp*x	(Leu, Val, Ile)x*x
Common Substrates	αII-spectrin 280 kDa	αII-spectrin 280 kDa
	βII-spectrin 260 kDa	βII-spectrin 260 kDa
Fragments Produced by	SBDP110 kDa	SBDP108 kDa
βII-spectrin	SBDP85 kDa	SBDP80 kDa
Cell Death Involvement	Most forms of necrosis	Most forms of apoptosis
	Some forms of apoptosis	

The table shows the different properties specific to calpain and caspase. Most importantly, it shows the consensus patterns at which each of the proteases cleaves. For caspase protease, the consensus pattern is DXXD and the cleavage site is right after this pattern (D is Aspartic Acid and X is any amino acid). Calpain, on the other hand, cleaves after the consensus patterns LX, VX, or IX, where L is Leucine, V is Valine, and I is Isoleucine.

**Table 2:**
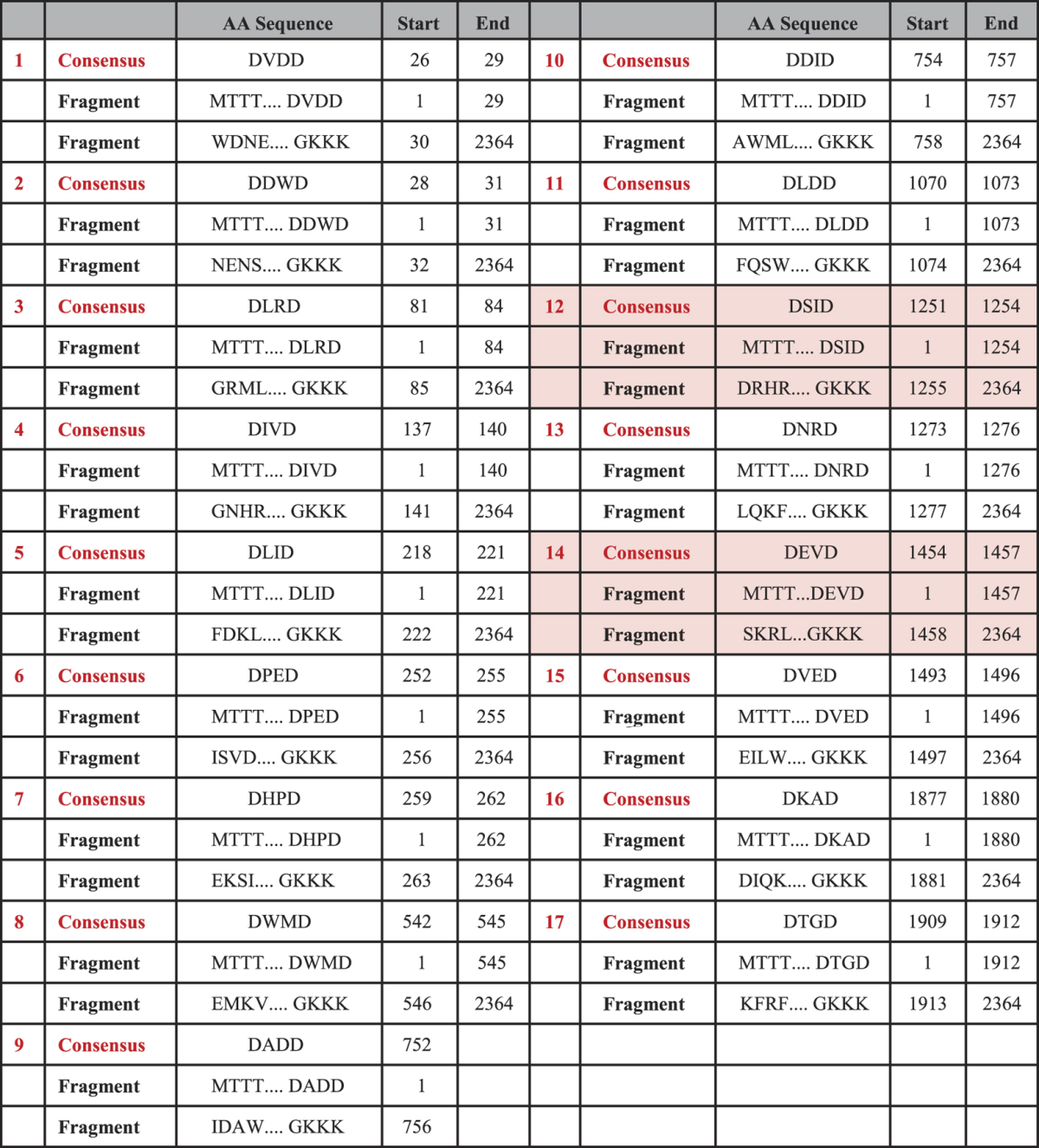
CFPA-CalpCasp Generated Data on M96803 (βII-spectrin Protein Sequence) by Caspase-3.

Table 2 shows the consensus occurrences detected by CFPA-CalpCasp algorithm, specific to caspase protease. Accordingly, it shows the detected pattern obeying DXXD (D is Aspartic Acid and X is any amino acid) and its start and end positions within the βII-spectrin protein sequence. Afterwards, the algorithm generates all the fragments resulting from caspase cleavage based on the corresponding consensus occurrence. The fragments are listed with their start and end positions within the βII-spectrin protein sequence. Red highlights show the consensus occurrences and the corresponding cleavages that are validated experimentally.

**Table 3:**
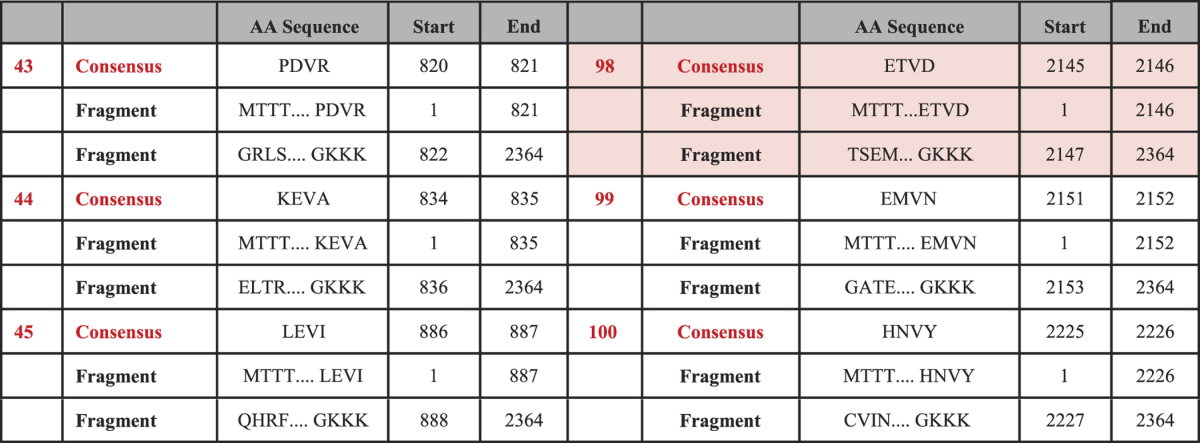
Few Records of CFPA-CalpCasp Generated Data on M96803 (βII-spectrin Protein Sequence) by Calpain-2 (see Supplementary Table 1 for all Output Records).

Table 3 shows the consensus occurrences detected by CFPA-CalpCasp algorithm, specific to calpain protease. Accordingly, it shows *few* detected patterns obeying VX (V is Valine and X is any amino acid) and its start and end positions within the βII-spectrin protein sequence. Afterwards, the algorithm generates all fragments resulting from calpain cleavage based on the corresponding consensus occurrence. The fragments are listed with their start and end positions within the βII-spectrin protein sequence. Red highlights show the consensus occurrence and the corresponding cleavage that are validated experimentally.

**Table 4:** Input Protein Sequence with Multiple Consensus Occurrences including Overlaps.

Input Protein Sequence (Seq. # 194)				
MLQDSITGIVNSFNLFFPSTMSRPTLMPTCVAFCSILFLTLATGCQAFPKVERRETAQEYAEKEQSQKMNTDDQENISFAPKYMLQQMSSEAPMVLSEGPSEIPLIKVFSVNKESHLPGAGLLHPTSPGVYSSSEPVVSASEQEPGPSLLERMSSEHSLSKVMLTVAVSSPASLNPDQEGPYNSLSTQPIVAAVTDVTHGSLDYLDNQLFAAKSQEAVSLGNSPSSSINTKEPEIIKADAAMGTTVVPGVDSTGDMEPDRERPSEMAADDGQSTTTKYLVTIPNNFLTTEPTAGSILGDAKVTVSVSTAGPVSSIFNEEWDDTKFESISRGRPPEPGDNAETQMRTKPPHGTYESFEGTEESPSSTAVLKVAPGHLGGEPALGTALVTALGDERSPVLTHQISFTPMSLAEDPEVSTMKLFPSAGGFRASTQGDRTQLSSETAFSTSQYESVPQQEAGNVLKDITQERKMATQAMNTTSPVVTQEHMATIEVPRGSGEPEEGMPSLSPVPAEVADAELSRRGESLATPASTTVVPLSLKLTSSMEDLMDTITGPSEEFIPVLGSPMAPPAMTVEAPTISSALPSEGRTSPSISRPNTAASYGLEQLESEEVEDDEDEEDEEDEEEEEEDEEDEEDEEDKETDSLYKDFDGDTEPPGFTLPGITSQEPDIRSGSMDLLEVATYQVPETIEWEQQNQGLVRSWMEKLKDKAGYMSGMLVPVGVGIAGALFILGALYSIKVMNRRRRNGFKRHKRKQREFNSMQDRVMLLADSSEDEF				

**Sequence Number**	**Consensus Occurrence #**	**Consensus**	**Start**	**End**
194	1	DEED	616	619
	2	DEED	619	622
	3	DEED	629	632
	4	DEED	632	635
	5	DEED	635	638

Table 4 shows a sample protein sequence from the whole mouse genome. This specific protein sequence is a sound case showing two functionalities implemented within the algorithm. The first is the multiple occurrences of the same consensus sequence (DEED) within the same protein sequence. The second is the overlapping consensus occurrences illustrated by their start and end positions (first DEED ends at 619 and next DEED starts at 619).
