# Supplementary material for: Novel Bioinformatics–Based Approach for Proteomic Biomarkers Prediction of Calpain-2 & Caspase-3 Protease Fragmentation: Application to βII-Spectrin Protein
Source: Sci Rep. 2017 Jan 23;7:41039. doi: 10.1038/srep41039 (PMC5253643; doi:10.1038/srep41039)
Supplement: Supplementary Information [file srep41039-s1.pdf]

Manuscript:
Novel Bioinformatics–Based Approach for Proteomic Biomarkers Prediction of Calpain-2 & Caspase-3 Protease Fragmentation: Application to βII-Spectrin Protein

Authors:
Atlal El-Assaad, Zaher Dawy, Georges Nemer, and Firas Kobeissy

```
>BetaII-spectrin from GeneBank M96803
MTTTVATDYDNIEIQQQYSDVNNRWDVDDWDNENSSARLFERSRIKALADEREAVQKKTFTKWVNSHLARVSCRITDLYTDLRD
GRMLIKLLEVLSGERLPKPTKGRMRIHCLENVDKALQFLKEQRVHLENMGSHDIVDGNHRLTLGLIWTIILRFQIQDISVETED
NKEKKSAKDALLLWCQMKTAGYPNVNIHNFTTSWRDGMAFNALIHKHRPDLIDFDKLKKSNAHYNLQNAFNLAEQHLGLTKLLD
PEDISVDHPDEKSIITYVVTYYHYFSKMKALAVEGKRIGKVLDNAIETEKMIEKYESLASDLLEWIEQTIIILNNRKFANSLVG
VQQQLQAFNTYRTVEKPPKFTEKGNLEVLLFTIQSKMRANNQKVYMPREGKLISDINKAWERLEKAEHERELALRNELIRQEKL
EQLARRFDRKAAMRETWLSENQRLVSQDNFGFDLPAVEAATKKHEAIETDIAAYEERVQAVVAVARELEAENYHDIKRITARKD
NVIRLWEYLLELLRARRQRLEMNLGLQKIFQEMLYIMDWMDEMKVLVLSQDYGKHLLGVEDLLQKHTLVEADIGIQAERVRGVN
ASAQKFATDGEGYKPCDPQVIRDRVAHMEFCYQELCQLAAERRARLEESRRLWKFFWEMAEEEGWIREKEKILSSDDYGKDLTS
VMRLLSKHRAFEDEMSGRSGHFEQAIKEGEDMIAEEHFGSEKIRERIIYIREQWANLEQLSAIRKKRLEEASLLHQFQADADDI
DAWMLDILKIVSSSDVGHDEYSTQSLVKKHKDVAEEIANYRPTLDTLHEQASALPQEHAESPDVRGRLSGIEERYKEVAELTRL
RKQALQDTLALYKMFSEADACELWIDEKEQWLNNMQIPEKLEDLEVIQHRFESLEPEMNNQASRVAVVNQIARQLMHSGHPSEK
EIKAQQDKLNTRWSQFRELVDRKKDALLSALSIQNYHLECNETKSWIREKTKVIESTQDLGNDLAGVMALQRKLTGMERDLVAI
EAKLSDLQKEAEKLESEHPDQAQAILSRLAEISDVWEEMKTTLKNREASLGEASKLQQFLRDLDDFQSWLSRTQTAIASEDMPN
TLTEAEKLLTQHENIKNEIDNYEEDYQKMRDMGEMVTQGQTDAQYMFLRQRLQALDTGWNELHKMWENRQNLLSQSHAYQQFLR
DTKQAEAFLNNQEYVLAHTEMPTTLEGAEAAIKKQEDFMTTMDANEEKINAVVETGRRLVSDGNINSDRIQEKVDSIDDRHRKN
RETASELLMRLKDNRDLQKFLQDCQELSLWINEKMLTAQDMSYDEARNLHSKWLKHQAFMAELASNKEWLDKIEKEGMQLISEK
PETEAVVKEKLTGLHKMWEVLESTTQTKAQRLFDANKAELFTQSCADLDKWLHGLESQIQSDDYGKHLTSVNILLKKQQMLENQ
MEVRKKEIEELQSQAQALSQEGKSTDEVDSKRLTVQTKFMELLEPLNERKHNLLASKEIHQFNRDVEDEILWVGERMPLATSTD
HGHNLQTVQLLIKKNQTLQKEIQGHQPRIDDIFERSQNIVTDSSSLSAEAIRQRLADLKQLWGLLIEETEKRHRRLEEAHRAQQ
YYFDAAEAEAWMSEQELYMMSEEKAKDEQSAVSMLKKHQILEQAVEDYAETVHQLSKTSRALVADSHPESERISMRQSKVDKLY
AGLKDLAEERRGKLDERHRLFQLNREVDDLEQWIAEREVVAGSHELGQDYEHVTMLQERFREFARDTGNIGQERVDTVNHLADE
LINSGHSDAATIAEWKDGLNEAWADLLELIDTRTQILAASYELHKFYHDAKEIFGRIQDKHKKLPEELGRDQNTVETLQRMHTT
FEHDIQALGTQVRQLQEDAARLQAAYAGDKADDIQKRENEVLEAWKSLLDACESRRVRLVDTGDKFRFFSMVRDLMLWMEDVIR
QIEAQEKPRDVSSVELLMNNHQGIKAEIDARNDSFTTCIELGKSLLARKHYASEEIKEKLLQLTEKRKEMIDKWEDRWEWLRLI
LEVHQFSRDASVAEAWLLGQEPYLSSREIGQSVDEVEKLIKRHEAFEKSAATWDERFSALERLTTLELLEVRRQQEEEERKRRP   &
PSPEPSTKVSEEAESQQQWDTSKGEQVSQNGLPAEQGSPRMAETVDTSEMVNGATEQRTSSKESSPIPSPTSDRKAKTALPAQS
AATLPARTQETPSAQMEGFLNRKHEWEAHNKKASSRSWHNVYCVINNQEMGFYKDAKTAASGIPYHSEVPVSLKEAVCEVALDY
KKKKHVFKLRLNDGNEYLFQAKDDEEMNTWIQAISSAISSDKHEVSASTQSTPASSRAQTLPTSVVTITSESSPGKREKDKEKD
KEKRFSLFGKKK
```

Supplementary Figure 1: βII-spectrin encoded gene. The figure shows the FASTA amino acid sequence of BetaII-spectrin from the GeneBank. This protein sequence is used as input to the algorithm CFPA-CalpCasp, which finds all consensus occurrences in it. Subsequently, the algorithm cuts the input sequence at the cleavage sites, generating all different fragments or breakdown products. http://www.ncbi.nlm.nih.gov/nuccore/338442/

```
Cleaved Fragments Prediction Algorithm for Calpain-2 and Caspase-3 (CFPA-CalpCasp)
Algorithm:
Input: N Protein Sequences, N' Consensus Sequences
Output: Occurrences and Fragments with Start and End Positions

BEGIN

While Not End Of Input Protein Sequences
    While Not End Of Consensus Sequences
        Run SW Algorithm on Cons_Seq and In_Seq
        For All Paths tracked
            While Not End Of Path
                If an INDEL is encountered
                {Reject Path, Modification to SW Alg.}
                        Set Path ← Skip-Path
                End If
            End While
            If the Path is a Skip-Path
                Continue
            Else Reset to Start of Path
            End If
            While Not End Of Path-O(m)
                If a Mismatch is encountered
                    Path_Mism_Cnt ← Path_Mism_Cnt +1
                End If
            End While
            {Accept Path, Modification to SW Alg}
            If (Path_Mism_Cnt == 0)
                Add Cons. Occ. node to Occ_Linked_List
                Update node w/ Occ. Details
                {Compute Consensus Occurrence Cut}
                Cut ← Path_Index – 1
                {Add to vector of all Cuts on Protein Sequence}
                Add Cut to vector Cuts
                Occ_Cnt ← Occ_Cnt + 1
            End If
        End For
        {Generate Fragments based on Occ_Cnt}
        For Poss_Occ_Cnt ← 1 to Occ_Cnt
            Gen_Frags(Poss_ Occ_Cnt, In_Seq)
        End For
    End While {All Input Protein Sequences}
End While{All Consensus Sequences}

Gen_Frags(In: Poss_ Occ_Cnt, In_Seq)
    {There is a vector for each combination generated}
    If Poss_ Occ_Cnt ← 0 {Exit Cond. of Recur. Funct.}
        Sort Comb. vector w/ 1 Combination of Cuts
        Gen. Frags. for a specific Comb. of Cuts
        Add each Fragment to Frags_Linked_List
        Output all Frags. per specific Comb. of Cuts
    Else
        {Gen. new Comb. based on Poss_Occ_Cnt value}
        For i ← 0 to (Cuts.Size - Poss_ Occ_Cnt)
            Push into Comb. vector the Value of Cuts[i]
            Gen_Frags(Poss_ Occ_Cnt - 1, In_Seq)
            Pop last element from Comb. vector
        End For
    End If

END
```

Supplementary Figure 2: CFPA-CalpCasp algorithm. The figure shows in detail the algorithm developed to detect all consensus occurrences. Once found, those occurrences enable both of caspase and calpain to cleave the substrate at the right cleavage site. The consensus is DXXD for caspase, where D is Aspartic Acid and X is any amino acid. For calpain, there exist more than one consensus pattern; they are LX, VX, or IX, where L is Leucine, V is Valine, and I is Isoleucine. Cleavage occurs right after all identified consensus occurrences.
